# What Is Learned in Pavlovian Conditioning in Crickets? Revisiting the S-S and S-R Learning Theories

**DOI:** 10.3389/fnbeh.2021.661225

**Published:** 2021-06-11

**Authors:** Makoto Mizunami

**Affiliations:** Faculty of Science, Hokkaido University, Sapporo, Japan

**Keywords:** classical conditioning, octopamine, dopamine, US devaluation, invertebrate, insect, evolution, cognition

## Abstract

In Pavlovian conditioning in mammals, two theories have been proposed for associations underlying conditioned responses (CRs). One theory, called S-S theory, assumes an association between a conditioned stimulus (CS) and internal representation of an unconditioned stimulus (US), allowing the animal to adjust the CR depending on the current value of the US. The other theory, called S-R theory, assumes an association or connection between the CS center and the CR center, allowing the CS to elicit the CR. Whether these theories account for Pavlovian conditioning in invertebrates has remained unclear. In this article, results of our studies in the cricket *Gryllus bimaculatus* are reviewed. We showed that after a standard amount of Pavlovian training, crickets exhibited no response to odor CS when water US was devalued by providing it until satiation, whereas after extended training, they exhibited a CR after US devaluation. An increase of behavioral automaticity by extended training has not been reported in Pavlovian conditioning in any other animals, but it has been documented in instrumental conditioning in mammals. Our pharmacological analysis suggested that octopamine neurons mediate US (water) value signals and control execution of the CR after standard training. The control, however, diminishes with extension of training and hence the CR becomes insensitive to the US value. We also found that the nature of the habitual response after extended Pavlovian training in crickets is not the same as that after extended instrumental training in mammals concerning the context specificity. Adaptive significance and evolutionary implications for our findings are discussed.

## Introduction

Pavlovian (or classical) conditioning, first reported by Pavlov in 1902 (Pavlov, 1927), refers to a learning process in which pairing of a biologically significant stimulus (unconditioned stimulus, US) with a relatively neutral stimulus (conditioned stimulus, CS) results in the CS eliciting a response (conditioned response, CR). Usually, the CR is similar to the response elicited by the US. Pavlovian conditioning is a basic form of associative learning ubiquitous among many vertebrates and invertebrates. Elucidation of questions such as what are the underlying neural mechanisms, what is its adaptive significance, what is learned during learning or what kind of associations underlie learned behavior is a fundamental issue of behavioral neuroscience. In this regard, insects have provided useful experimental animals to investigate basic neural mechanisms of Pavlovian conditioning and its adaptive significance (Menzel, 2012). For example, in the fruit-fly *Drosophila melanogaster*, the use of advanced transgenic technologies allowed detailed analysis of neural and molecular mechanisms of Pavlovian conditioning, and it has been demonstrated that neural circuits of the mushroom bodies, highly organized multisensory associative centers of the insect brain, play critical roles for achieving conditioning (Hige, 2017; Eschbach et al., 2020; Modi et al., 2020). Adaptive significance of Pavlovian conditioning, as well as its cost (such as decreased longevity associated with increased capability of long-term memory formation in the fruit-fly, Lagasse et al., 2012), has been examined in some insects including the grasshopper *Schistocerca americana* (Dukas and Bernays, 2000) and the fruit-fly (Mery and Kawecki, 2005; Lagasse et al., 2012). However, the question about the nature of associative processes governing the CR has received little attention until very recently in insects. In this review, I briefly summarize our attempts to characterize associative processes that account for the CR in crickets and propose that associations that are formed by conditioning and govern the CR in crickets are fundamentally similar to those in mammals.

## Associations That Govern Pavlovian Conditioned Responses in Mammals: S-S and S-R Theories

**FIGURE 1:**
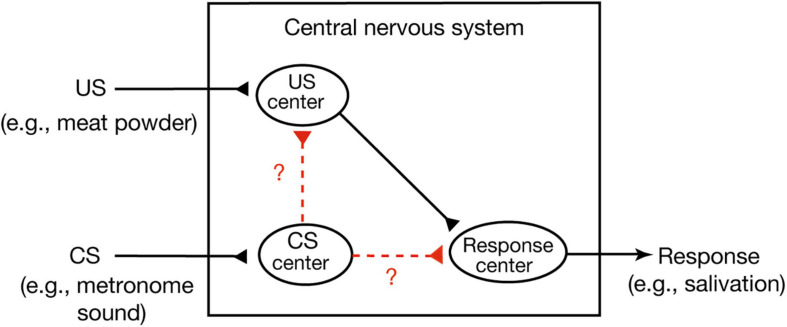
Two possible versions of Pavlovian conditioning. In Pavlovian conditioning, neural connection or association may develop from the CS center to the US center or from the CS center directly to the response center. The former matches S-S association theory and the latter matches S-R association theory (Mazur, 2017). Pavlov’s stimulus substitution theory (Pavlov, 1927) can be considered as a form of the S-S learning theory.

A widely held view of conditioned behavior in higher vertebrates (birds and mammals) is that animals learn an association between the CS and internal representation of the US and that the CR is produced because the CS activates an internal representation of the US (Mazur, 2017). This theory is called the stimulus-stimulus (S-S) learning theory. An example of this is Pavlov’s stimulus substitution theory (Pavlov, 1927). He assumed that there are three centers, a US center, a CS center and a CR center, in the central nervous system (Figure 1; Mazur, 2017). The first or the second is activated when a US or CS is presented, respectively, and activation of the third elicits a CR. He proposed that conditioning forms a new association or connection between the CS center and the US center, which is termed a stimulus-stimulus (S-S) association. An alternative view, called the S-R learning theory, is that conditioning establishes a new association or connection between the CS center and the CR center, a stimulus-response (S-R) association (Mazur, 2017). Formation of such a direct sensorimotor pathway has been reported in Pavlovian conditioning of gill withdrawal response in the sea hare *Aplysia* (Kandel, 2001). In this conditioning, paired presentations of a strong stimulus to the tail (US) and a gentle tactile stimulus to the siphon (CS) elicit an enhancement of efficacy of synaptic transmission from CS-responding interneurons to motoneurons that produce gill withdrawal response (CR). Hence, CS elicits the CR after conditioning.

A procedure widely used for discrimination of the S-S type learning and S-R type learning is a test of the effect of devaluation of the US on execution of the CR. In the case of conditioning of sound CS with food US in rats, for example, rats receive pairing of a CS and a US in a training box and then receive devaluation of the US, either by providing the food until satiation or by taste aversion learning for associating the food with a harmful toxin (Holland and Rescorla, 1975), and then the amount of general activity during CS presentation is tested as a measure of CR. If the CR is reduced by US devaluation, it can be considered that the CR is guided by representation of the current value of the US, in accordance with the S-S theory. On the other hand, if the CR is unaffected by US devaluation, the CR is considered to be independent of the US value, in accordance with the S-R theory. CRs that are sensitive to US devaluation have been found in a wide range of conditioning systems in mammals, including conditioning of a sound with food in rats described above (Holland and Rescorla, 1975). CRs that are insensitive to US devaluation have also been found in several conditioning preparations (Holland, 2008). An example is a behavior referred to as sign-tracking behavior in rats, in which rats approach and contact the lever after receiving conditioning of a lever with food (Nasser et al., 2015). In invertebrates, however, little effort has been made to investigate which of these two theories better accounts for the CR.

## Pavlovian Conditioning in Crickets

**FIGURE 2:**
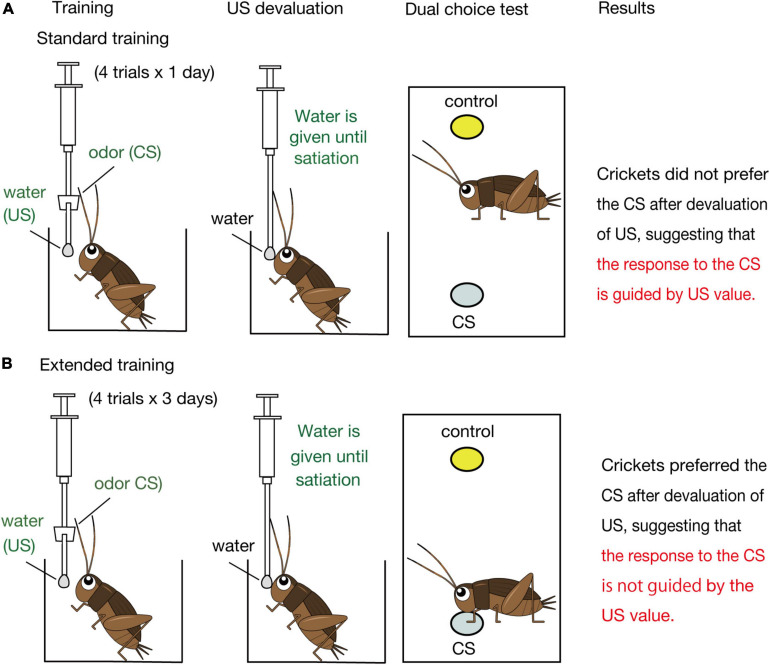
Schematic illustration of the effects of US devaluation on execution of a CR after standard **(A)** and extended **(B)** Pavlovian training in crickets. Crickets that had received standard training (4-trial × 1-day training) or extended training (4-trial × 3-day training) to associate an odor CS with water US were given water until they stopped drinking prior to the post-training test (Mizunami et al., 2019). In the test, crickets that had received standard training exhibited no significant preference for the CS over a control odor (i.e., did not spend a significantly longer time for exploring at the CS odor source than at the control odor source), whereas crickets that had received extended training exhibited a significant preference for the CS over the control odor (i.e., spent a significantly longer time for exploring at the CS odor source), as did control crickets that received no US devaluation before the test.

Matsumoto and Mizunami (2002) developed a simple but effective procedure for Pavlovian conditioning in the cricket *Gryllus bimaculatus*, in which an odor is paired with water as appetitive US or a high concentration of sodium chloride solution as aversive US (Figure 2, left). A cricket is placed in a beaker and deprived of water for 3 days. A syringe containing water or sodium chloride is used for conditioning. A small filter paper soaked with an essence of CS odor or control odor is attached to the needle of the syringe. For conditioning, the filter paper is approached to the cricket’s antennae for 3 s and then a drop of water or sodium chloride solution is attached to the mouth. The effect of conditioning is evaluated by testing relative preference between the CS odor and a control odor before and after conditioning (Figure 2, right). In the test, a cricket is placed in an arena, on the floor of which there are two containers that contain a filter paper soaked with an essence of CS odor or control odor, covered with a gauze net. Relative time that the cricket spent touching the top net of the odor sources with palpi or antennae is measured, and a change of relative time before and after training is used as a measure of CR. We use the exploratory behavior at the CS odor source as CR since it is analogous to exploratory behavior at a water source. We referred to this procedure as a “classical conditioning and operant testing procedure,” which is based on a high capability of crickets to transfer memory formed in a classical conditioning situation to an operant testing situation (Matsumoto and Mizunami, 2002; Unoki et al., 2005, 2006).

We observed that a single trial to associate an odor with water or sodium chloride solution is sufficient to achieve altered odor preference when tested 30 min after the training (Unoki et al., 2005). In appetitive conditioning with water US, two to four pairing trials with 5-min inter-trial intervals are sufficient to produce protein synthesis-dependent memory that lasts at least 4 days, which matches the standard definition of long-term memory (Matsumoto and Mizunami, 2002; Matsumoto et al., 2003). In aversive conditioning with sodium chloride US, 6 trials are needed for establishing long-term memory (Unoki et al., 2005).

Subsequent pharmacological studies by Unoki et al. (2005, 2006) and Mizunami et al. (2009) using octopamine (OA) receptor antagonists (such as epinastine) and dopamine (DA) receptor antagonists (such as flupentixol) suggested that aminergic neurons play critical roles for conditioning and for execution of the CR. Injection of saline containing epinastine into the head haemolymph at 30 min prior to appetitive conditioning of an odor with water impaired conditioning, whereas injection of flupentixol did not impair this conditioning. In contrast, flupentixol impaired aversive conditioning of an odor with salt water, but epinastine had no effect (Unoki et al., 2005, 2006). Moreover, injection of epinastine at 30 min prior to the post-training test impaired execution of appetitive CR, whereas injection of flupentixol did not impair it. In contrast, flupentixol impaired execution of aversive CR, but epinastine had no effect (Mizunami et al., 2009). We thus suggested that octopamine (OA) neurons, which are considered as the invertebrate counterpart of noradrenaline neurons (Roeder, 1999, but see also an alternative view by Bauknecht and Jékely, 2017), are activated by the presentation of an appetitive US and that their activation is necessary for appetitive conditioning and for execution of the appetitive CR. Similarly, we suggested that dopamine (DA) neurons are activated by the presentation of an aversive US and that their activation is necessary for aversive conditioning and for execution of the aversive CR (Unoki et al., 2005, 2006; Nakatani et al., 2009; Mizunami et al., 2009; Matsumoto et al., 2015; Mizunami and Matsumoto, 2017). Studies with knockdown or knockout of genes that code OA or DA receptors by the RNAi or Crispr/cas9 technique confirmed critical roles of OA and DA neurons in appetitive and aversive conditioning, respectively (Awata et al., 2015, 2016).

Terao et al. (2015) subsequently investigated stimulus conditions that are necessary for achieving conditioning, and observed a learning phenomenon called “blocking,” which was first discovered in rats by Kamin (1969). In mammals, blocking has been best accounted for by error-correction learning theories, according to which conditioning is governed by the prediction error, i.e., the discrepancy between the US that an animal receives and the US that the animal predicts to receive (Domjan, 2015; Mazur, 2017). Terao et al. (2015) observed blocking and a specific case of blocking, “one-trial blocking,” and suggested that Pavlovian conditioning in crickets is best accounted for by the Rescorla and Wagner (1972) model, one of most influential models among error-correction learning theories that are proposed to account for Pavlovian conditioning. Moreover, our pharmacological studies suggested that OA neurons mediate prediction error signals for appetitive conditioning (Terao et al., 2015), whereas DA neurons mediate prediction error signals for aversive conditioning (Terao and Mizunami, 2017; Mizunami et al., 2018), although evidence for the latter is incomplete. These suggestions are comparable to findings in mammals that different types of DA neurons in the midbrain mediate appetitive and aversive prediction error signals, respectively, in Pavlovian conditioning as well as in instrumental conditioning (Schultz, 2013, 2015; Engelhard et al., 2019; Gershman and Uchida, 2019). Thus, we suggested that Pavlovian conditioning in crickets is based on learning rules that are fundamentally similar to those in mammals (Mizunami et al., 2018).

**FIGURE 3:**
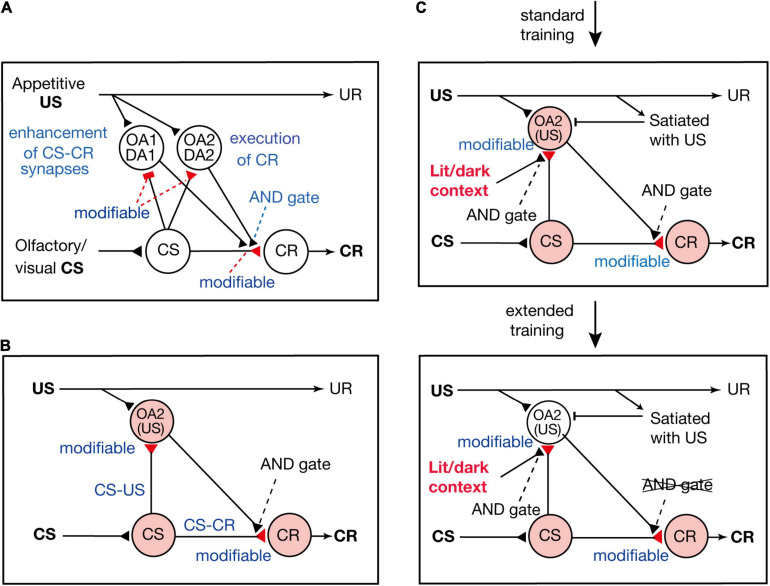
Models of appetitive Pavlovian conditioning in crickets that account for the change from a US value-sensitive CR to a habitual one with extension of training. **(A)** A model of Pavlovian conditioning (Terao et al., 2015) that consists of four types of neurons: “CS” neurons that code the CS, two classes of OA or DA neurons (“OA1/DA1” neurons and “OA2/DA2” neurons) that code appetitive/aversive US, and “CR” neurons that produce the CR. The “OA1/DA1” neurons or “OA2/DA2” neurons receive inhibitory or excitatory synapses from “CS” neurons, the efficacy of which is strengthened by pairing of the CS and the US and by resulting simultaneous activation of their pre- and postsynaptic neurons (assuming Hebbian plasticity). The efficacy of excitatory synapses from “CS” neurons to “CR” neurons is strengthened when “CS” neurons and “OA1/DA1” neurons are activated at the same time (assuming Kandelian plasticity, Kandel, 2001), and “CR” neurons are activated when “CS” neurons and “OA2/DA2” neurons are activated at the same time (shown as AND gate). **(B)** A part of the model is shown for highlighting the roles of “OA2” neurons for execution of appetitive CR. **(C)** In this model, we revised our previous model in **(B)** to account for the finding that the CR is sensitive to US devaluation after standard training but not after extended training (Mizunami et al., 2019) and that the CR is initially specific to the condition of illumination under which the cricket received training, but the specificity is lost after extended training (Sato et al., 2021). We assume that activation of “OA2” neurons does not occur when the animal is satiated with the US or when the test is performed outside the context of training. Hence, a CR does not occur after US devaluation or outside the context of training after standard training. We also assume that the efficacy of “CS-CR” synapses is further enhanced by extended training, so that activation of “CR” neurons occurs without activation of “OA2” neurons, and hence the CR occurs after US devaluation or outside the context of training. **(A,B)** Modified from Mizunami et al. (2018). **(C)** Modified from Sato et al. (2021).

Terao et al. (2015) proposed a neural circuit model of Pavlovian conditioning in crickets (Figure 3A), which is assumed to represent the neural circuit of mushroom bodies. The model consists of four types of neurons: “CS” neurons that mediate CS signals, “CR” neurons that receive excitatory synapses from “CS” neurons and their activation produces a CR, and two types of OA or DA neurons that are activated by appetitive or aversive US and make synapses with axon terminals of “CS” neurons. One of the two types of OA or DA neurons (“OA1/DA1” neurons) governs conditioning and receives inhibitory synapses from “CS” neurons, whereas the other type (“OA2/DA2” neurons) governs execution of a CR and receives excitatory synapses from “CS” neurons. There are three assumptions in the model. The first assumption is that synaptic transmission from “CS” neurons to “OA1/DA1” neurons and that from “CS” neurons to “CR” neurons are enhanced by coincident activation of “CS” neurons and “OA1/DA1” neurons. The second assumption is that synapses from “CS” neurons to “OA2/DA2” neurons are enhanced by coincident activation of their pre- and postsynaptic neurons. The third assumption is that coincident activation of “CS” neurons and “OA2/DA2” neurons is needed after conditioning to activate “CR” neurons and to produce a CR.

According to the model proposed by Terao et al. (2015), presentation of a CS after conditioning activates both the “CS-OA2/DA2” pathway and the “CS-CR” pathway, and coincident activation of both pathways activates “CR” neurons and produces a CR. Therefore, in our model, both the S-S and S-R pathways are formed by conditioning and are activated for execution of a CR (see Figure 3B); our model is thus characterized as an S-S and S-R hybrid model.

## CR Is Sensitive to Us Devaluation After Standard Training But Not After Extended Training

Mizunami et al. (2019) then asked how such presumable dual associative structures influence the nature of the CR regarding sensitivity to US devaluation. We focused on appetitive conditioning and the roles of OA neurons in execution of appetitive CR (Figure 3B) since devaluation of appetitive US is easier than that of aversive US. Crickets were water-deprived for 3 days and were subjected to a standard amount of training (4 pairing trials with 5-min inter-trial intervals, which we refer to as standard training or 4-trial × 1-day training, Figure 2A). One day after training, they were given water until satiation. In a subsequent test, the crickets exhibited no significant level of preference for the conditioned odor over a control odor. Control experiments showed that the loss of preference for the CS is not because water satiation reduced sensory or motor function or motivation necessary to explore odor sources. Therefore, we concluded that crickets do not respond to a CS when crickets are satiated with the US. We thus suggested that the CR is guided by US expectancy, as expected by the S-S learning theory.

Recent studies have shown that CRs in other species of insects are also sensitive to US devaluation. A study of olfactory conditioning in honey bees showed a significant reduction of the CR by devaluation of sucrose US by pairing it with quinine, indicating that the CR contains a devaluation-sensitive component (Lai et al., 2020). A study of olfactory conditioning with sucrose or water US in the fruit-fly *Drosophila* also showed a significant reduction of responses to sucrose- or water-associated CS when the flies were satiated with the US (Senapati et al., 2019). Therefore, S-S type learning in which a CR occurs depending on the current value of the US is not rare in insects.

In fruit flies, in which it has been shown that dopamine (DA) neurons mediate sucrose or water US signals for appetitive conditioning (Liu et al., 2012), optogenetic activation of a specific type of DA neurons after conditioning of an odor with water or sucrose reward produces a CR in hungry or thirsty flies but not in sated flies (Huetteroth et al., 2015). These findings are consistent with our model in crickets. It needs to be investigated whether such US-mediating neurons are activated during execution of a CR.

Mizunami et al. (2019) observed, on the other hand, that crickets that received extended training exhibit a normal level of CR after US devaluation (Figure 2B). Crickets that received 4 trials of training each day on three consecutive days (4-trial × 3-day training) and then received US devaluation prior to the test significantly preferred the conditioned odor over a control odor. This finding indicates that the response to the CS occurs independently of the US value, in accordance with the S-R learning theory. We thus concluded that the CR is initially controlled by the current value of the US but that the control is lost with extension of training in crickets. To our knowledge, a loss of sensitivity of a CR to US devaluation by extended training has not been reported in Pavlovian conditioning in mammals (Holland, 1998, 2005; Holland et al., 2008; Keefer et al., 2020) or in any other animals.

In order to investigate conditioning parameters that are necessary to make the CR insensitive to US devaluation, we performed 12-trial × 1-day training and 6-trial × 2-day training, the number of trials being the same as 4-trial × 3-day training, and we tested the effect of US devaluation on the CR (Mizunami et al., 2019). We observed that the CR is sensitive, at least in part, to US devaluation in these trainings, indicating that these trainings are not sufficient to make the CR fully independent of the US value. The results suggest that a larger number of trainings *per se* is not the reason for the CR becoming independent of the US value. Rather, repetitive trainings with sufficiently long intervals are necessary to make the CR insensitive to US devaluation.

## Neural Circuit Model for Formation of a Habitual CR by Extended Training

Mizunami et al. (2019) proposed a model to account for the loss of sensitivity of the CR to US devaluation by extended training. In the model (Figure 3C), we added two new assumptions to our previous model (Figure 3B; Terao et al., 2015). The first new assumption is that activation of “OA2” neurons is inhibited when animals are satiated with the US and the second assumption is that the requirement of activation of “OA2” neurons for production of a CR is lost after extended training. A possible reason for the latter is that the efficacy of “CS-CR” synapses is further strengthened by extended training, so that “CR” neurons can be activated by activation of “CS” neurons alone without activation of “OA2” neurons. In short, the model assumes that CS-induced activation of “OA2” neurons controls the execution of the CR early in training but that the control is lost after extended training. In other words, the CR early in training is based on activation of both the S-S pathway and the S-R pathway (Figure 3B), but it is based solely on activation of the S-R pathway after extended training.

The model predicts that administration of an OA receptor antagonist prior to the post-training test abolishes the CR after standard training but that it has no effect after extended training. The results of our pharmacological study were in accordance with this prediction (Mizunami et al., 2019). We also examined whether conditioning parameters that are necessary to make the CR insensitive to administration of an OA receptor antagonist match the conditioning parameters that are necessary to make the CR insensitive to US devaluation. We observed that the CR is abolished at least in part by administration of an OA receptor antagonist in 12-trial × 1-day training and 6-trial × 2-day training. This finding is in accordance with our finding that the CR is diminished at least in part by US devaluation in these trainings and is hence in agreement with the model. It should be cautioned, however, that the model is a conceptual one and how it is implemented in actual neural circuits of the cricket brain needs to be investigated by physiological studies.

## A Shift From Devaluation-Sensitive Responses to Devaluation-Insensitive Responses Is Also Found After Extended Instrumental Training in Mammals

Interestingly, a change from the initial actions that are sensitive to reward devaluation to responses that are insensitive to reward devaluation with the progress of training has been documented in instrumental conditioning in mammals (Dickinson, 1985; Yin and Knowlton, 2006; Smith and Graybiel, 2014). In instrumental conditioning of lever pressing for obtaining food in rats, for example, lever-pressing actions early in training are in a large part sensitive to devaluation of food reward and hence governed by expectancy of outcome of the instrumental behavior, but actions after extended training are in a large part insensitive to reward devaluation and hence independent of outcome expectancy (Dickinson, 1985; Yin and Knowlton, 2006; Smith and Graybiel, 2014). It should be cautioned, however, that the change is not a change in an all-or-none manner, i.e., both goal-directed and habitual response components are present both early in training and after extended training (Dickinson, 1985; Yin and Knowlton, 2006; Smith and Graybiel, 2014). Devaluation-insensitive responses after extended instrumental training in mammals have been termed habitual responses. Following this terminology, we refer to devaluation-insensitive responses after extended Pavlovian training in crickets as habitual responses.

Mizunami et al. (2019) proposed an updated conceptual definition of formation of a habitual response by extended training so that it can be used in both instrumental conditioning and Pavlovian conditioning. It has been argued that learned action early in instrumental training in mammals depends mainly on the action-outcome (A-O) association but that the action becomes dependent more on the stimulus-response (S-R) association with the progress of training (Dickinson, 1985; Yin and Knowlton, 2006; Smith and Graybiel, 2014). In Pavlovian conditioning in crickets, our model shown in Figure 3C indicates that execution of the CR requires activation of both the S-S and S-R associations early in training but that it becomes dependent solely on the S-R association after extended training. Thus, formation of a habitual response by extended training can be defined as learned behavior becoming dependent more on the S-R association in both Pavlovian conditioning and instrumental conditioning.

## Reduced Context Specificity of the CR After Extended Training

Sato et al. (2021) then investigated whether a habitual (devaluation-insensitive) response after extended Pavlovian training in crickets has features analogous to those of a habitual response after extended instrumental training in mammals. In instrumental conditioning in rats, it has been well established that habitual behavior that is insensitive to outcome devaluation is characterized by higher context specificity, i.e., the response is less likely to occur outside the context in which training is performed (Thrailkill and Bouton, 2015), in which the context is defined as the physical surrounding, state or time. The same has been demonstrated in instrumental learning in humans (Gardner, 2015; Wood and Rünger, 2016).

We performed standard or extended training in crickets under illumination and tested the CRs under illumination or in the dark 1 day later (Sato et al., 2021). We found that crickets that had received standard training (4-trial × 1-day training) under illumination exhibited a higher level of CR under illumination than that in the dark. On the other hand, crickets that had received extended training (4-trial × 3-day training) under illumination exhibited the same levels of CR under illumination and in the dark. Thus, the CR is initially context-specific, but it loses context specificity with the extension of training. In our model, this can be accounted for, for example, by assuming that synaptic transmission from “CS” neurons to “OA2” neurons is gated by neurons that mediate signals about context (Figure 3C). In this case, “OA2” neurons are not activated outside the training and hence a CR does not occur early in training, but the CR occurs outside the training context after extended training since activation of “OA2” neurons is no longer required for producing a CR. In conclusion, the influential notion that habitual behavior after repetitive training is more context-specific in instrumental learning in mammals including humans (Gardner, 2015; Wood and Rünger, 2016) does not apply to Pavlovian conditioning in crickets. The reasons for the difference remain to be investigated.

## Functional and Evolutionary Considerations

I conclude that different training protocols lead to CRs of different natures, i.e., a CR that is governed by the current value of the US and is based on an S-S association or a CR that is independent of the US value and is based on an S-R association in crickets. CRs that are sensitive to US devaluation and those that are insensitive are found in Pavlovian conditioning systems in mammals (Holland, 2008; Clark et al., 2012). It should be asked what is the functional significance for having two types of CRs, each being based on either the S-S or S-R associative mechanism. The CR guided by the US value allows flexible adjustment of learned behavior in accordance with the current requirement of the animal, whereas a more automatic or habitual CR allows the cognitive function of the brain to be used for other tasks. For the former, the response guided by representation (or memory) of the US value has another advantage in that it allows new learning. Mizunami et al. (2009) investigated second-order conditioning in crickets, in which after conditioning of a CS (CS1) with an appetitive or aversive US, CS1 is paired with another CS (CS2). This results in conditioning of CS2 with the US. Our pharmacological analysis suggested that CS1 presentation in the second training stage activates OA or DA neurons that code appetitive or aversive US signals and that this activation produces conditioning of the CS2 with the appetitive or aversive US (Mizunami et al., 2009, see also Matsumoto et al., 2013). This is analogous to the finding of “CS-mediated learning” in rats (Holland, 1998, 2005; Holland et al., 2008), in which after conditioning of a CS with food US, conditioning of the CS with an aversive toxin results in aversion to the food, presumably because CS presentation in the second training stage activates representation of food US, and this activation produces conditioning of food with the toxin.

With a closer look at the CR, however, distinctions of the nature of the CR between the Pavlovian conditioning system in crickets and the systems in mammals are evident, in that a shift from a goal-directed CR to a habitual (devaluation-insensitive) one by extended training has not been reported in any systems of Pavlovian conditioning in mammals. Formation of habitual responses by extended training is a well-established feature of instrumental conditioning in mammals, but the nature of their habitual responses differed from those formed in Pavlovian conditioning in crickets as we have discussed. Such a difference may reflect different evolutionary histories of Pavlovian conditioning systems in mammals and insects.

**FIGURE 4:**
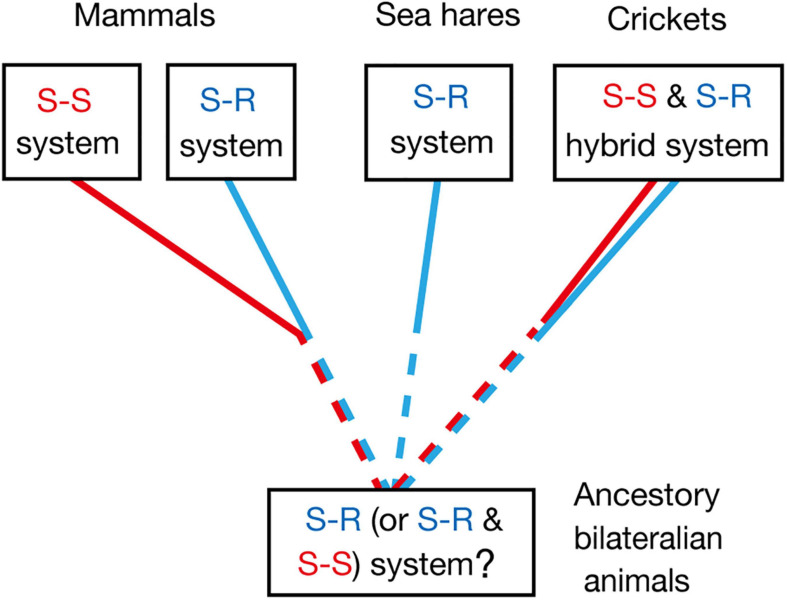
Evolutionary considerations for the S-R type and S-S type Pavlovian conditioning systems, which can be discriminated by US devaluation. Pavlovian conditioning of gill withdrawal response in the sea hare *Aplysia* can be judged as S-R type from its neural circuitry mechanisms (Kandel, 2001). Pavlovian conditioning systems in mammals and birds are in many cases an S-S type, though S-R type conditioning systems are also present (Holland, 2008; Mazur, 2017). Olfactory conditioning in crickets can be characterized as an S-S and S-R hybrid type. Pavlovian conditioning has been reported in planarians (Prados et al., 2013), which are evolutionary-basal bilaterian animals. Investigations of the effect of US devaluation on execution of a CR in planarians and other invertebrates are needed for obtaining more insights into the evolution of Pavlovian conditioning systems.

Common ancestors of insects and mammals are thought to be bilaterian invertebrate animals that are phylogenetically close to flatworms (Sarnat and Netsky, 2002). Pavlovian conditioning has been demonstrated in planarians, which are flatworms (Prados et al., 2013), and hence it can be speculated that the common ancestors had the capability of Pavlovian conditioning. Whether Pavlovian conditioning in planarians is based on the S-R or S-S type learning mechanism, or its hybrid, is unknown, and this needs to be clarified for obtaining insights into the evolution of Pavlovian conditioning systems. The most plausible possibility is that it is based on the S-R type learning system, since S-S type learning may require well-organized associative networks that allow a CS to activate internal representation or memory of the CS-associated US, such as insect mushroom bodies, but such highly organized neuropils have not been observed in the head ganglia of planaria (Sarnat and Netsky, 2002; Cebrià, 2008). Nevertheless, the possibility that S-S type learning also emerged in very early stage in evolution of Pavlovian conditioning systems should not be easily dismissed (Figure 4).

The capability of Pavlovian conditioning can be considered an important cognitive tool shared by many vertebrates and invertebrates that enabled animals to predict future events and to adapt their behavior to changes in the environment. Further elaboration of the Pavlovian conditioning system into the S-S associative learning system allowed animals to adjust their behavior in accordance with the changes of their specific needs for the US. Such sophistication has been achieved in mammals, birds and insects and probably in many other groups of animals. Further studies on Pavlovian conditioning in various animal groups are needed to elucidate how this fundamental cognitive function has been elaborated in different lineages of animals.

## Author Contributions

MM wrote the manuscript.

## Conflict of Interest

The author declares that the research was conducted in the absence of any commercial or financial relationships that could be construed as a potential conflict of interest.
